# Sudden onset anhidrosis in an otherwise healthy male

**DOI:** 10.1002/ski2.242

**Published:** 2023-05-15

**Authors:** Clara Michelle Tan Hwei Sian Tan, Rayson Lee, Janet Pei Ling Dua

**Affiliations:** ^1^ National University of Singapore Yong Loo Lin School of Medicine Singapore Singapore; ^2^ Tan Tock Seng Hospital Singapore Singapore; ^3^ Ng Teng Fong General Hospital Singapore Singapore

## Abstract

Acquired idiopathic generalised anhidrosis (AIGA) is a rare disorder that is characterised by sudden onset generalised absence of sweating without any dermatological, neurological or sweat gland abnormalities. AIGA predominately affects young males, mostly involving patients of Asian descent. There have been approximately 100 reported cases worldwide, most of which were reported in Japan. In Singapore, it is rarely seen with one case series on 15 cases of AIGA reported in a 2014 study. Here, we present a case of AIGA who responded well to conservative management with sweating activity.

## Graphical abstract

Sudden onset anhidrosis in an otherwise healthy 59‐year‐old Chinese male who presented to the dermatology department with a 3‐month history of a sudden lack of sweat production after picking up long distance cycling for 2 months

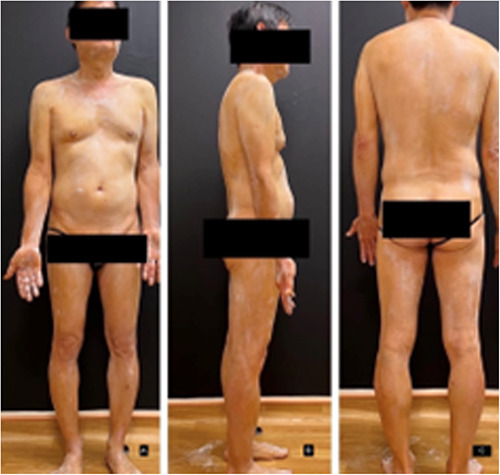

## 1 CASE REPORT

A 59‐year‐old Chinese male presented to the dermatology department with a 3‐month history of a sudden lack of sweat production after picking up long distance cycling for 2 months. He had previously never engaged in such strenuous exercise. The lack of sweating was associated with urticaria and feeling faint during exercise. The patient was otherwise fit and well with a unremarkable medical history of well‐controlled high cholesterol and gout for which he was taking simvastatin and allopurinol. He denied any previous skin inflammatory disease or any autoimmune disease. Laboratory investigations ordered revealed a normal IgE level.

**FIGURE 1:**
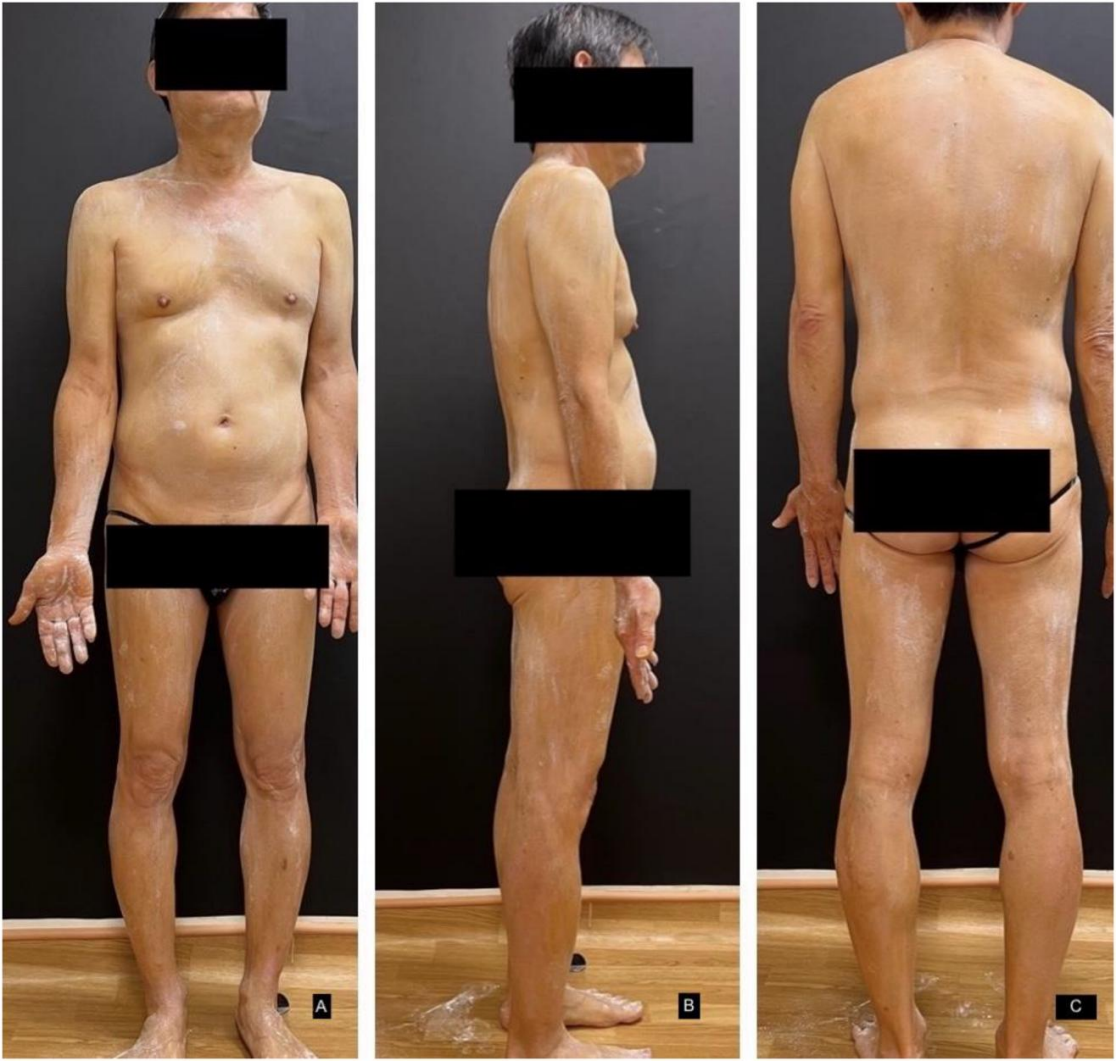
Minor starch‐iodine sweat test: before exercising for 40 min.

**FIGURE 2:**
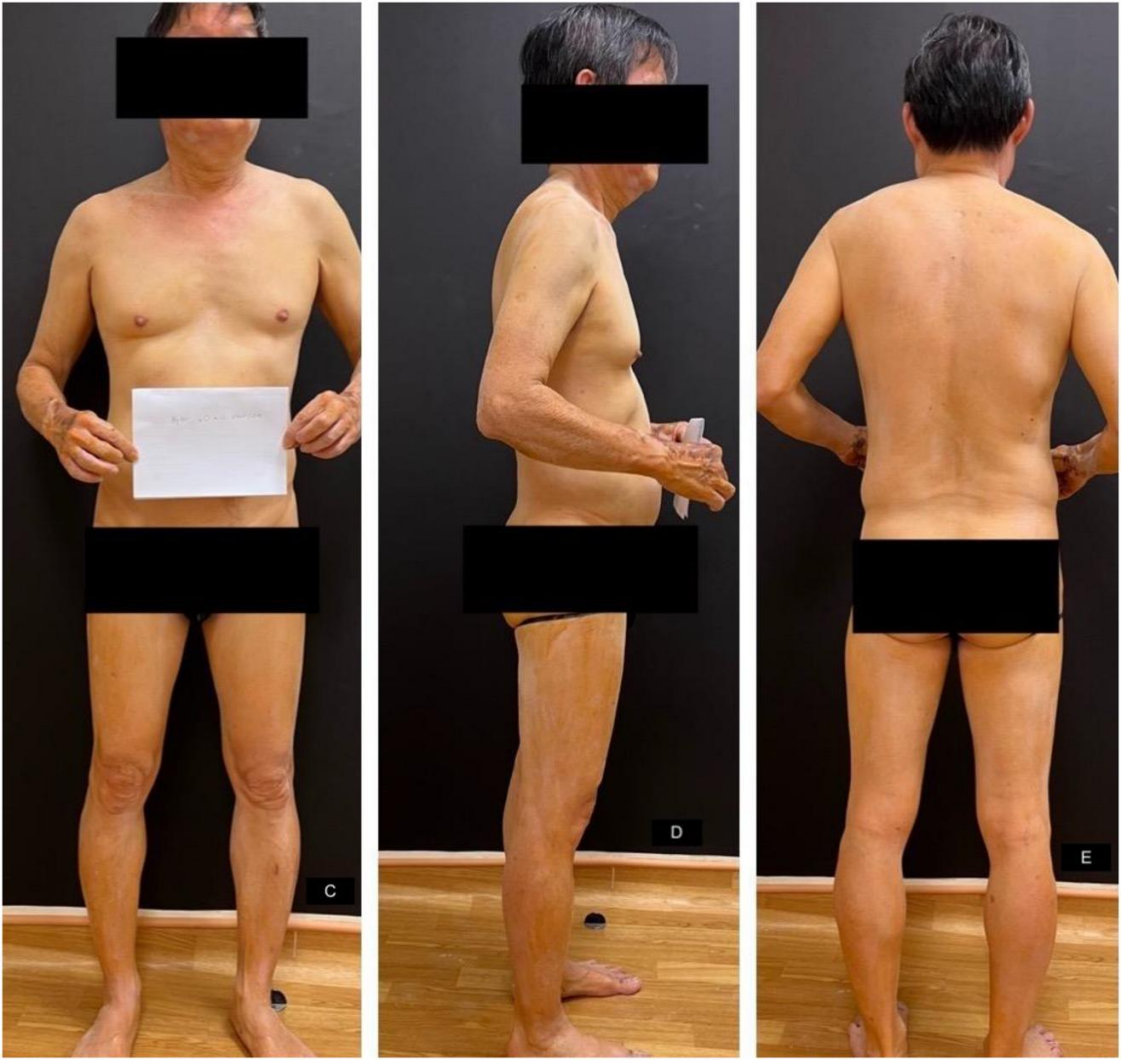
Minor starch‐iodine sweat test: after exercising for 40 min.

**FIGURE 3:**
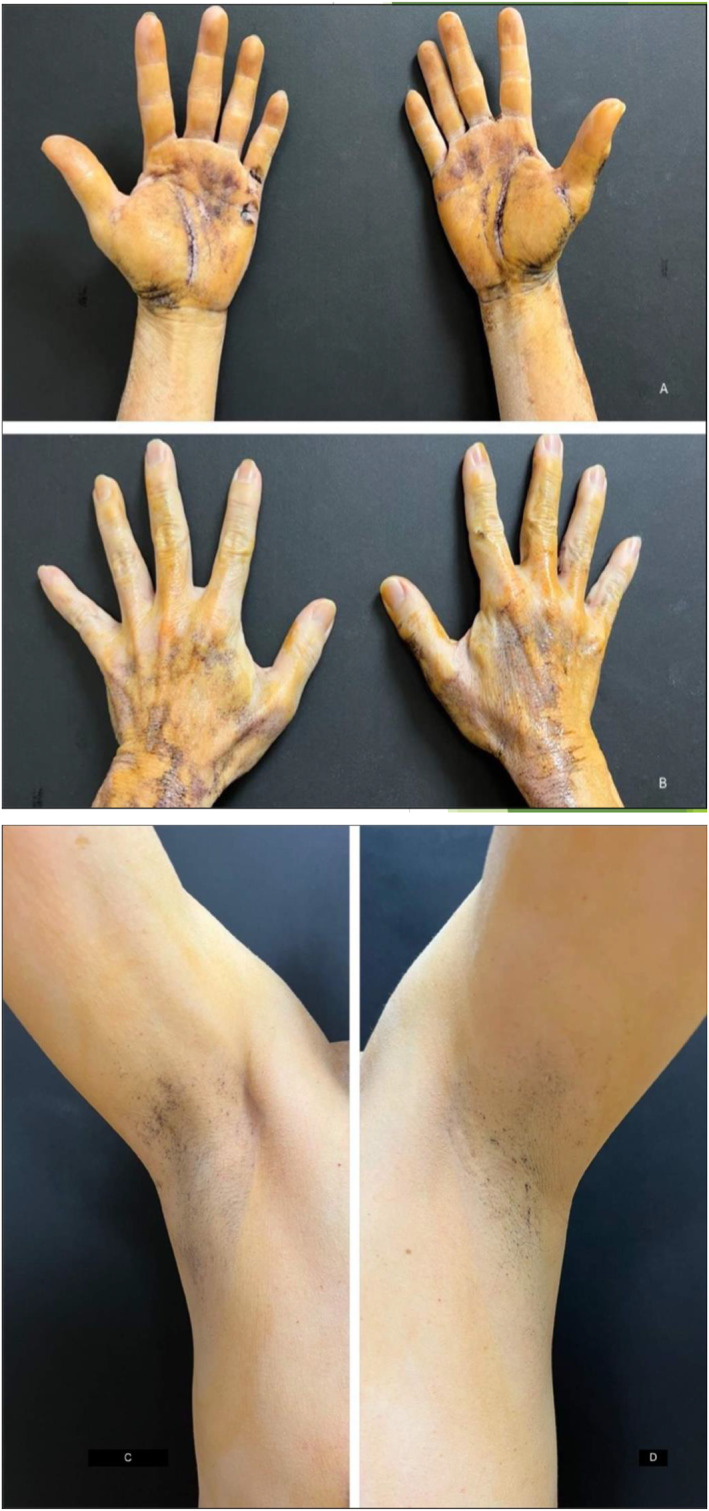
Dark blue colouration over the dorsum of his (a & b) hands and palms and (c & d) axilla.

A starch‐iodine sweat test was performed to confirm the extent of anhidrosis (Figure 1). After 40 min of exercise, the patient was revealed to have generalised anhidrosis sparing his hands and axilla as shown in (Figures 2 & 3).

After 4 months of follow‐up, our patient's anhidrosis spontaneously improved with conservative management. New areas of sweating were present over his arms and chest.

## 2 DISCUSSION

Anhidrosis can be spilt into either congenital or acquired. Congenital anhidrosis involves congenital alterations in the eccrine glands as seen in congenital anhidrotic diseases such as Hypohidriotic Ectodermal Dysplasia (HED), Congenital insensitivity to pain with anhidrosis (CIPA) or Fabry disease. On the other hand, acquired anhidrosis can be spilt into 2 subgroups: idiopathic anhidrosis or secondary anhidrosis. Secondary anhidrosis can be attributed to organic causes in the form of central or neuropathic disease as well as due to certain medications.^1^

In this case, due to absence of organic causes, this is a form of acquired idiopathic generalised anhidrosis (AIGA).

AIGA can be associated with 3 subtypes: sudomotor neuropathy, idiopathic pure sudomotor failure (IPSF) and sweat gland failure (SGF).^2^

Most cases of AIGA are categorised under idiopathic pure sudomotor failure (IPSF), whereby skin biopsies are either normal or have mild peri‐eccrine lymphocytic infiltrate. It is also characterised by diminished sweating with sudomotor stimuli characterised by hyperthermia, dry skin, itch or tingling of the skin surface.^3^

The pathophysiology of IPSF remains relatively unknown. However, autoimmune mechanisms targeting the CHRM3 (Cholinergic Receptor Muscarinic 3) receptors have been noted as a possible cause in recent literature. Their response to the binding of acetylcholine is inhibited, resulting in a lack of sweat response.^4^

In IPSF, only the eccrine sweat glands with cholinergic innervation are affected. Apocrine sweat glands of the axilla are under adrenergic control while eccrine sweat glands of the palms and soles are under both adrenergic and cholinergic control. Hence, this could be the reason for continued sweating over the palms and axilla region.

IPSF also commonly presents with cholinergic urticaria. Cholinergic Urticaria usually occurs during perspiration and involves acetylcholine. It was proposed that cholinergic urticaria can be categorised into 2 subtypes: acetylcholine‐indirectly induced, sweat allergic type and acetylcholine‐directly induced, depressed sweating type.^2^ Acetylcholine is known to induce degranulation and subsequently histamine release from the mast cells.^3^ In acetylcholine‐indirectly induced type, acetylcholine stimulates perspiration. Sweat ducts might be damaged or obstructed resulting in leakage of sweat antigens produced into the dermis, hence stimulating mast cells to release histamine.^2^

In contrast, in the acetylcholine‐directly induced type which is also recognized as Cholinergic Urticaria with anhidrosis/hypohidrosis, eccrine sweat glands lack the expression of CHRM3 receptors thus the expression of CHRM3 is absent in anhidrotic areas and slightly present in hypohydrotic areas. The possible pathophysiology behind this is that acetylcholine released cannot be trapped by the absent CHRM3 receptors, resulting in an increase in the levels of acetylcholine into adjacent mast cells. This subsequently results in mast cell degranulation hence the formation of wheals.^2^

There has been a case report on 2 patients with this subtype of anhidrosis experiencing sharp, pinprick pain over their body sparing their palms and soles, exacerbated by heat and exercise.^4^ Some patients with IPSF have also reported experiencing cholinergic urticaria and in some cases, a raised serum IgE.

In order to distinguish it from the 2 subtypes, skin biopsy should be done first, followed by other tests such as electromicroscopy or autonomic fibre marker vasoactive intestinal peptide.^5^ The lack of abnormalities in the nerve fibres and sweat glands allow it to be distinguished from the other 2 subtypes, sudomotor neuropathy and sweat gland failure.^4^

**FIGURE 4:**
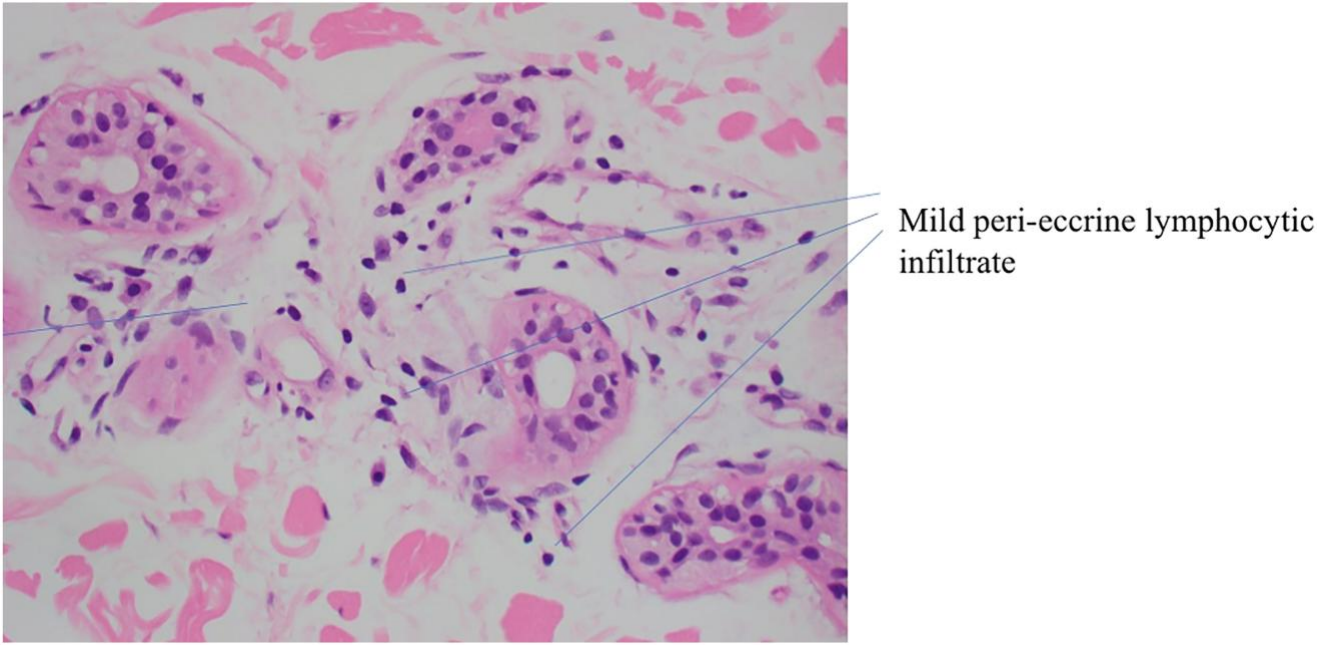
Patient's skin biopsy showing mild peri‐eccrine lymphocytic infiltrate with intact normal eccrine glands.

In this case, it is likely that our patient had the IPSF subtype due to the presence of normal eccrine sweat glands with mild peri‐eccrine infiltrates (Figure 4), sudden onset of symptoms with sweating over the hands and axilla only and cholinergic urticaria.

Regarding the possible triggers, sudden onset of anhidrosis has been similarly reported in young males undergoing mandatory national service training, and also in horses that have been trained strenuously under hot and humid conditions (equine anhidrosis).^6^ Similarly, in our patient, a sudden onset of such strenuous exercise could have precipitated his AIGA as he was not previously conditioned to strenuous activity.

Sweating is important in regulating human body temperature by dissipating thermal energy from skin surface when water in sweat evaporates.^7^ If not managed adequately, severe AIGA could result in the body temperature of these patients to increase to dangerous levels leading to heat stroke.^6^ This can also decrease the patient's quality of life due to the restrictions to the amount of exercise and outdoor activities the patient can engage in.

There is also evidence of association of AIGA with autoimmune diseases in the form of complications. Thus, it could be postulated that AIGA may be mediated via immunological and inflammatory mechanisms.^2^ In response to this mechanism, primary treatment of AIGA may consist of steroid pulse therapy or high dose oral steroids. The effectiveness of oral cyclosporine use has also been reported in a limited number of patients^8^ and is speculated to be due to an underlying potential autoimmune cause of IPSF. Other possible medications to be considered especially for patients with steroid resistant IPSF includes anti‐histamines which has reports of moderate success in some cases.^4^ This finding is supported in a recent case report which found that a type 1 histamine receptor antagonist is able to counter the inhibitory effect of histamine on acetylcholine‐induced sweating. However, classical sedative antihistamines that have an anticholinergic effect were said to be avoided because this may reduce acetylcholine‐induced sweating.^10^ Oral immunosuppressants can also be used in patients who are unresponsive to or are unable to use steroids.

In this case, spontaneous resolution occurred with conservative management.

It is equally important for the treatment of this condition to be anchored in good patient education.^9^ The disease should be explained to the patients so that they understand the importance of taking adequate precautions to prevent overheating especially in hot and humid climates. Only then, can we prevent life‐threatening consequences and maximise the patient's quality of life.

## CONFLICT OF INTEREST STATEMENT

None to declare.

## AUTHOR CONTRIBUTIONS

**Clara Michelle Tan Hwei Sian Tan:** Writing – original draft (Equal); Writing – review & editing (Equal). **Rayson Rui Sheng Lee:** Investigation (Equal); Methodology (Equal); Supervision (Equal); Writing – review & editing (Equal). **Janet Pei Ling Dua:** Data curation (Equal); Formal analysis (Equal); Investigation (Equal); Methodology (Equal); Supervision (Equal); Writing – review & editing (Equal)

## ETHICS STATEMENT

Not applicable.

## Data Availability

The data that support the findings of this study are available from the corresponding author upon reasonable request.
